# Ganglioneuroma of the Bladder in Association with Neurofibromatosis Type 1

**DOI:** 10.3390/diagnostics12123126

**Published:** 2022-12-12

**Authors:** Elena Ţarcă, Elena Cojocaru, Laura Mihaela Trandafir, Alina Costina Luca, Alina Sinziana Melinte Popescu, Lăcrămioara Ionela Butnariu, Marian George Melinte Popescu, Dana Teodora Anton Păduraru, Mihaela Moscalu, Daniela Rusu, Viorel Ţarcă

**Affiliations:** 1 Department of Surgery II-Pediatric Surgery, “Grigore T. Popa” University of Medicine and Pharmacy, 700115 Iaşi, Romania; 2 Department of Morphofunctional Sciences I–Pathology, “Grigore T. Popa” University of Medicine and Pharmacy, 700115 Iaşi, Romania; 3 Department of Mother and Child Medicine–Pediatrics, “Grigore T. Popa” University of Medicine and Pharmacy, 700115 Iaşi, Romania; 4 Department of General Nursing, Faculty of Medicine and Biological Sciences, “Ştefan cel Mare” University of Suceava, 720229 Suceava, Romania; 5 Department of Medical Genetics, Faculty of Medicine, “Grigore T. Popa” University of Medicine and Pharmacy, 700115 Iaşi, Romania; 6 Department of Preventive Medicine and Interdisciplinarity, ”Grigore T. Popa” University of Medicine and Pharmacy, 700115 Iaşi, Romania; 7 Department of Surgery, “Grigore T. Popa” University of Medicine and Pharmacy, 700115 Iaşi, Romania

**Keywords:** hematuria, neurofibromatosis, bladder ganglioneuroma

## Abstract

Neurofibromatosis type 1 (NF1) is a genetic disease, with autosomal dominant transmission, related to pathogenic variant of the tumor suppressor gene *NF1* (17q11.2), predisposing affected subjects to a variety of benign (neurofibromas and plexiform neurofibromas) and malignant tumors. The lack of the NF1-neurofibromin gene product can cause uncontrolled cell proliferation in the central or peripheral nervous system and multisystemic involvement, and so the disease includes a heterogeneous group of clinical manifestations. Ganglioneuromas are benign tumors developing from the neural crest cells of the autonomic nervous system, considered to be part of neuroblastic tumors. Bladder localization is extremely rare in adults, and only three such cases were reported in children so far. The aim of our study, in addition to a brief review of the literature of these pathologies, is to bring to your attention the case of a sixteen year old patient with a very rare association of NF1 and bladder ganglioneuroma, who presented at the hospital with gross hematuria. Since bladder ganglioneuroma is a rare pathological condition, the differential diagnosis is difficult and imaging investigations and pathological investigations are the ones that elucidate this disease. The clinical approach of the medical multidisciplinary team involved should help the patient in managing her medical and surgical situation.

## 1. Introduction

Neurofibromatosis type 1 (NF1; OMIM 613113) or Von Recklinghausen disease is an autosomal dominant genetic disease related to pathogenic variant of the tumor suppressor gene *NF1* (located on chromosome 17q11.2), predisposing affected subjects to a variety of benign (neurofibromas and plexiform neurofibromas) and malignant tumors. The incidence is around 1:3500 newborns [1]. Even though it is an autosomal dominant disease, over 50% of neurofibromatosis cases are due to a new (de novo) mutation, affecting any population regardless of race or sex [2].

The lack of the NF1-neurofibromin product, a peptide with tumor-suppressor action, can cause uncontrolled cell proliferation in the central or peripheral nervous system and multisystemic involvement (e.g., dermatological, cardiovascular, gastrointestinal, genitourinary, orthopedic, etc.), and so the disease includes a heterogeneous group of clinical manifestations. To confirm the diagnosis of type 1 neurofibromatosis in a patient without a parent with NF1, the presence of at least 2 of the following standardized clinical criteria is required: 1. Six or more café-au-lait macules over 5 mm in diameter in prepubertal persons or over 15 mm in post pubertal individuals; 2. Two or more typical neurofibromas or a plexiform neurofibroma; 3. Axillary or inguinal freckles (Crowe sign; lentiginous macules); 4. Distinct bone lesions; 5. Glioma of the optic nerve; 6. Two or more hamartomas of the iris (Lisch nodules) or two or more choroidal abnormalities; 7. Heterozygous pathogenic NF1 variant with a variant allele fraction of 50% in apparently normal tissue. If one parent is already diagnosed with neurofibromatosis, the child only needs one or more of the above criteria for being diagnosed with NF1. The recently revised criteria for NF1 incorporated new clinical features (choroidal anomalies) and genetic testing [3].

Ganglioneuromas are benign tumors developing from the neural crest cells of the autonomic nervous system, and are considered to be part of neuroblastic tumors (neuroblastoma, ganglioneuroblastoma, and ganglioneuroma). They can develop anywhere along the sympathetic nerve chain and are fully differentiated and benign [4]. Bladder localization is extremely rare in adults, and only three such cases have been reported in children so far [1,5,6].

### Justification of the Case Report

The aim of our study, in addition to a brief review of the literature of these pathologies, is to bring to your attention the case of a patient with a very rare association of neurofibromatosis type 1 and bladder ganglioneuroma.

## 2. Case Report

We present a female patient, S.M.C., diagnosed at the age of 16 with type 1 neurofibromatosis, meeting the 2021 revised NF1 diagnostic criteria: café au lait spots, axillary and inguinal freckling, dystrophic scoliosis, labial plexiform neurofibroma; she also had slight intellectual disability (learning problems). At the initial presentation she came with gross hematuria, dysuria, and abdominal pain. An abdominal ultrasound was performed and detected a tumor in the bladder; the imaging investigation was completed with an uro-computed tomography, cranial and spine magnetic resonance imaging (MRI). Subsequently, the patient was multidisciplinary investigated (genetic, cardiological, nephrological, neurological, neurosurgical, ophthalmological, neuropsychiatric, orthopedic, anatomopathological, radiological, endocrinological), performing a complex clinical-paraclinical assessment. Laboratory analyses showed anemia and urinary tract infection with *Enterococcus faecalis* and *Proteus mirabilis*. The patient received hemostatic treatment, antibiotics, analgesics, with a favorable evolution. After one month, she came back with abdominal pain and urethrocystoscopy was performed, followed by open tumor biopsy and partial excision of the bladder tumor. The pathological examination established the diagnosis of ganglioneuroma and then the patient received antibiotics for preventing urinary tract infection and analgesics. In this case, a major role in the management of her condition was also the psychological counseling at the periodic reassessments because she previously had suicidal attempts. At the last follow up, the patient was asymptomatic, the biological investigations and imaging investigations were showing improvement in her state, and she declared an improvement in the quality of life.

### 2.1. History and Clinical Findings

**Figure 1:**
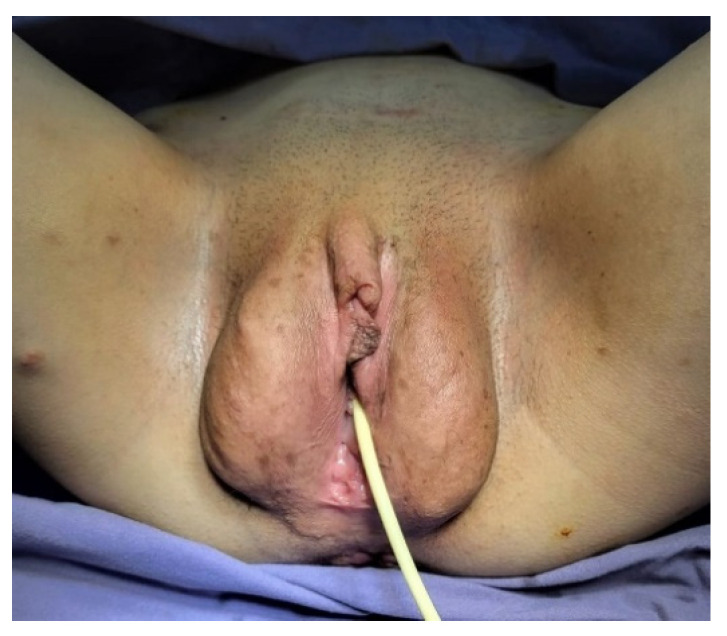
Hypertrophy of the labia majora (plexiform neurofibroma).

**Figure 2:**
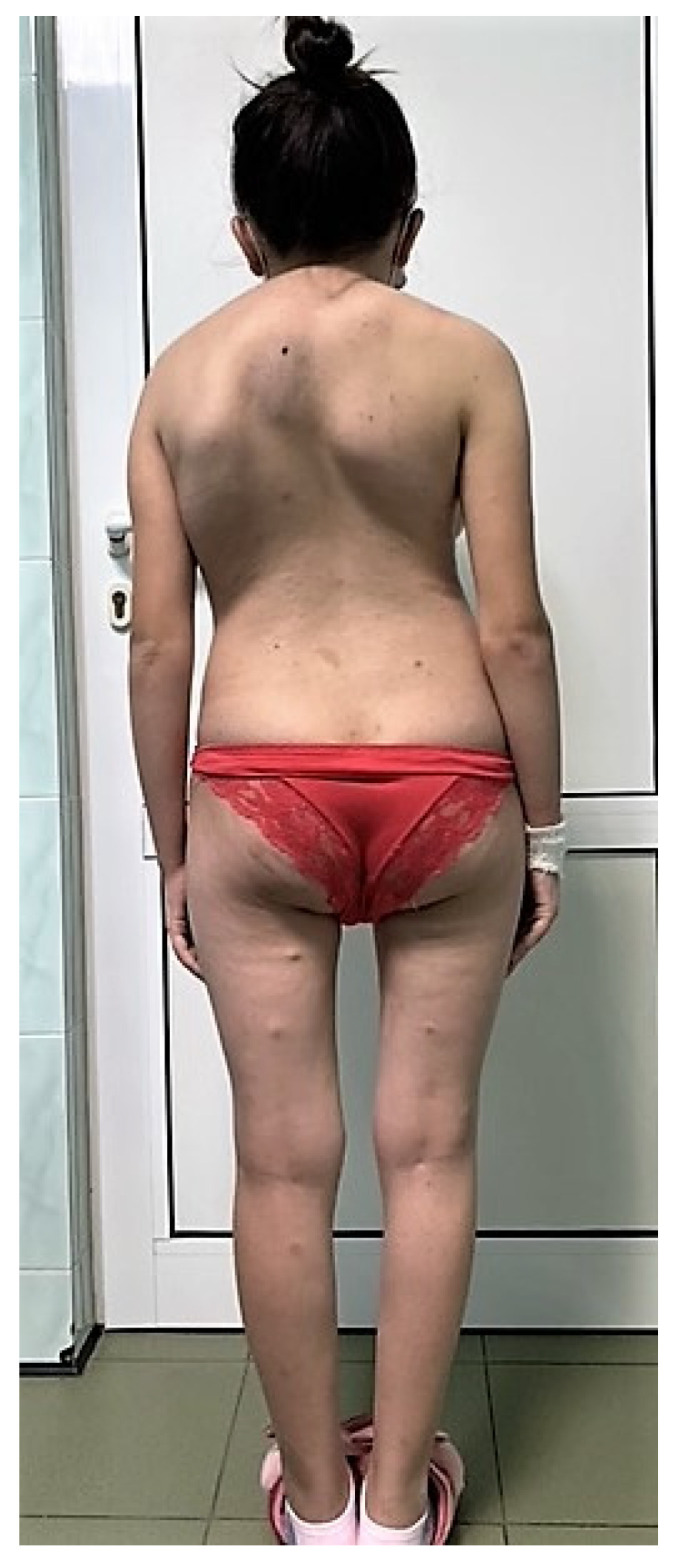
Short stature for age, subcutaneous nodules, and kyphoscoliosis.

A 16-year-old girl was hospitalized in our clinic for hematuria, dysuria, and urinary frequency symptoms that lasted for three months. Her medical history included multiple episodes of depression and a suicide attempts, having multiple autolytical scars on the anterior face of the left arm. The socio-economic environment from which the patient came was a disadvantaged one, the teenager being raised by an uncle, while the mother worked abroad. Although not investigated or diagnosed with NF1 before, the patient’s mother had multiple café-au-lait spots and subcutaneous neurofibromas. Physical examination of the patient revealed short stature for age, axillary and inguinal freckles, and multiple café-au-lait spots around her body; she also had more than two subcutaneous nodules and hypertrophy of the labia majora (plexiform neurofibroma) (Figure 1). No neurologic deficiency was observed in her examination, although she has severe kyphoscoliosis (Figure 2). The ophthalmological consultation revealed the absence of Lisch nodules and amblyopia in the left eye.

The psychological examination revealed emotional disorders and borderline intellectual and learning difficulties.

### 2.2. Imaging Findings

**Figure 3:**
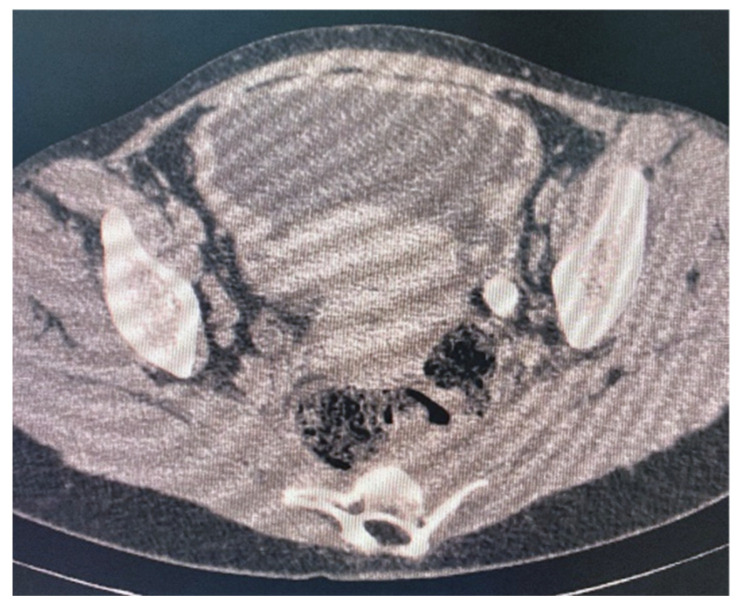
Uro-CT showing an infiltrative contrast enhancing mass located at the level of the posterior bladder wall.

**Figure 4:**
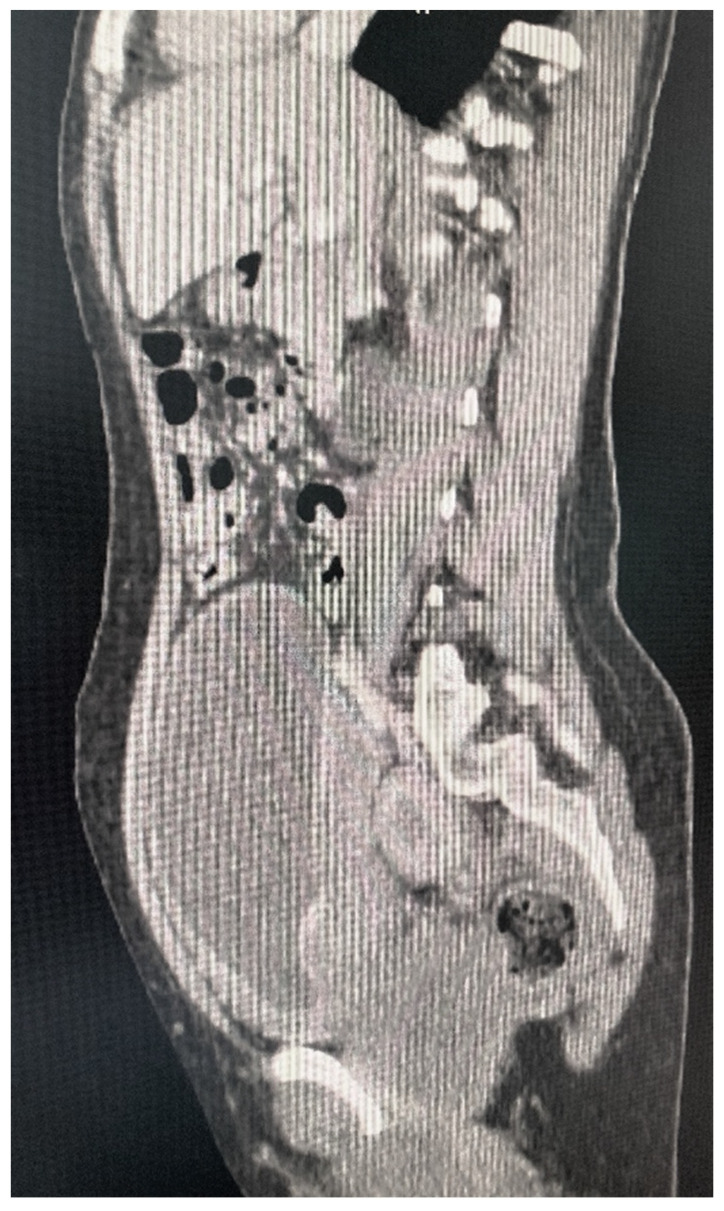
Uro-CT–lateral view.

Abdominal ultrasound: a large tumor with wide implantation base (84.8/38 mm) was seen in the urinary bladder at the level of the posterior wall, with intense Doppler signal and irregular and pseudo polypoid internal contour. Computerized tomography with urography (Uro-CT) showed infiltrative, moderate increases in density with enhanced CT scan mass located at the level of the posterior bladder wall, dimensions 22.2/87.4/95 mm; thickening of the lower bladder wall up to 9.2 mm and infiltration of the right uretero-vesical junction; bilateral grade II ureterohydronephrosis (Figure 3 and Figure 4). Cranial and spine CT and MRI revealed normal optic chiasm; important left-convex dorsal scoliosis (76° Cobb) with axial rotation of the vertebral bodies; vertebral bodies D5, D6 wedge-shaped, with osteophytes and disc changes.

**Figure 5:**
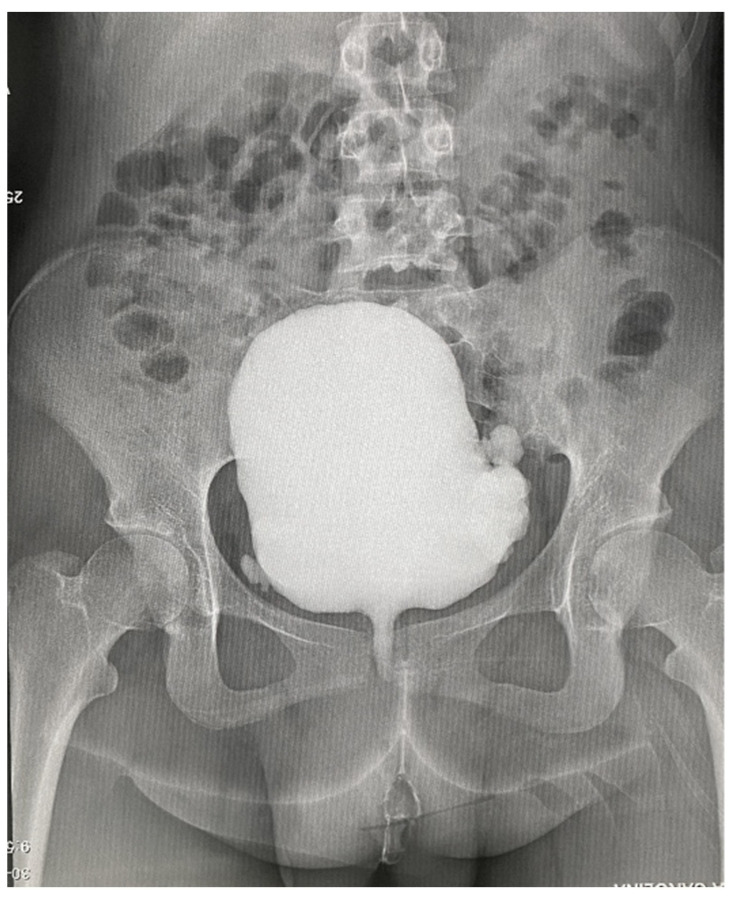
Micturition urethrocystography showing vesical diverticula.

One year after surgical intervention, micturition urethrocystography was performed, which visualized the bladder with an irregular outline due to the presence of addition images (vesical diverticula) with variable sizes at the side walls of the bladder; absence of vesicoureteral reflux (Figure 5).

### 2.3. Operative and Pathological Findings

**Figure 6:**
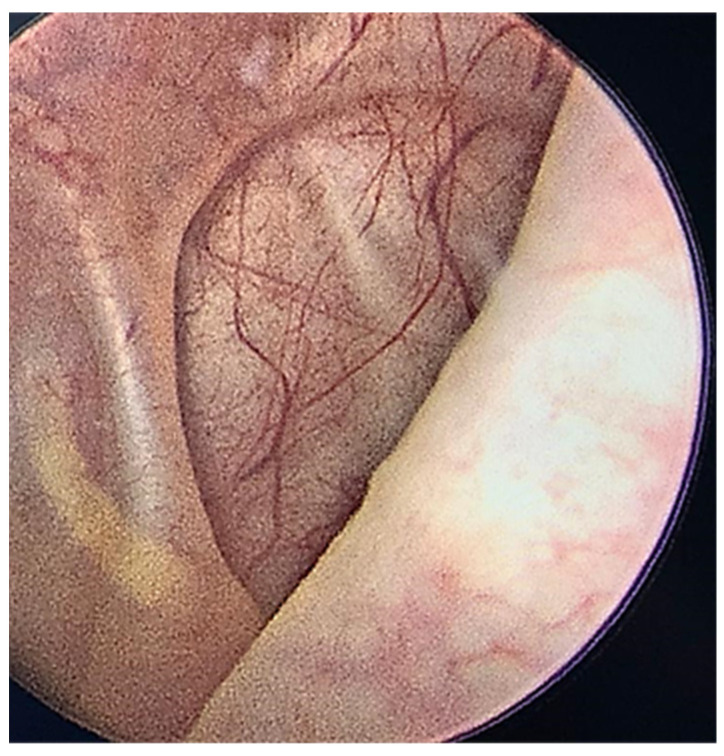
“Fighting” appearance of the bladder wall, with “cells and columns”.

Urethrocystoscopy revealed the presence of the bladder tumor as well as the “fighting” appearance of the bladder wall with “cells and columns” and diverticula on the lateral walls (Figure 6). Tissue samples and a partial removal of the tumor were obtained with open resection of the polypoid mass invading the posterior wall of the bladder. The pathologic examination revealed a bladder ganglioneuroma.

**Figure 7:**
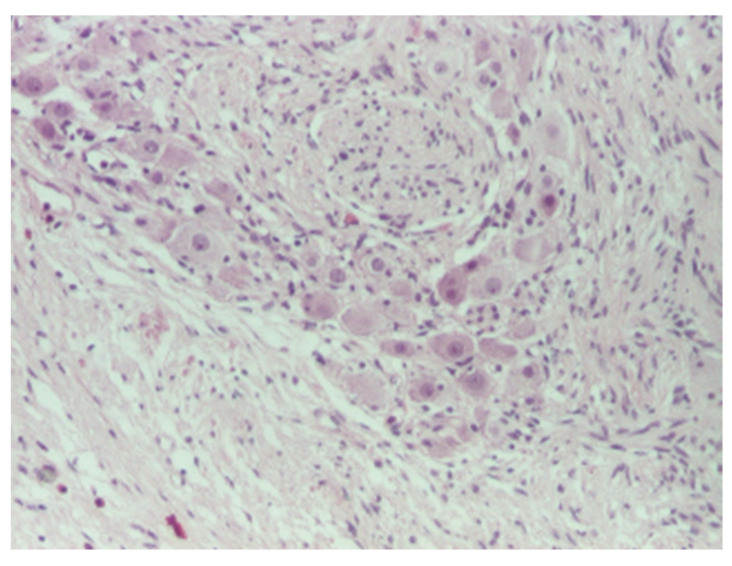
Mature ganglion cells admixed with stroma, HE × 100.

**Figure 8:**
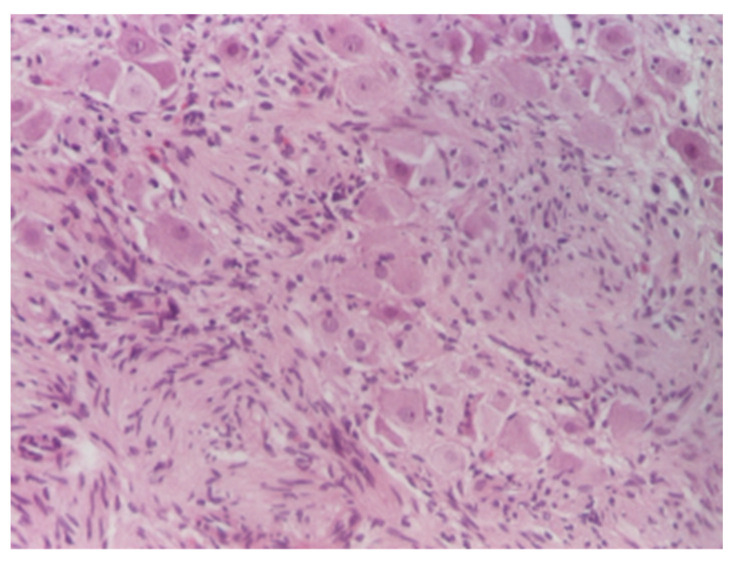
Scattered mature ganglion cells, HE ×100.

**Figure 9:**
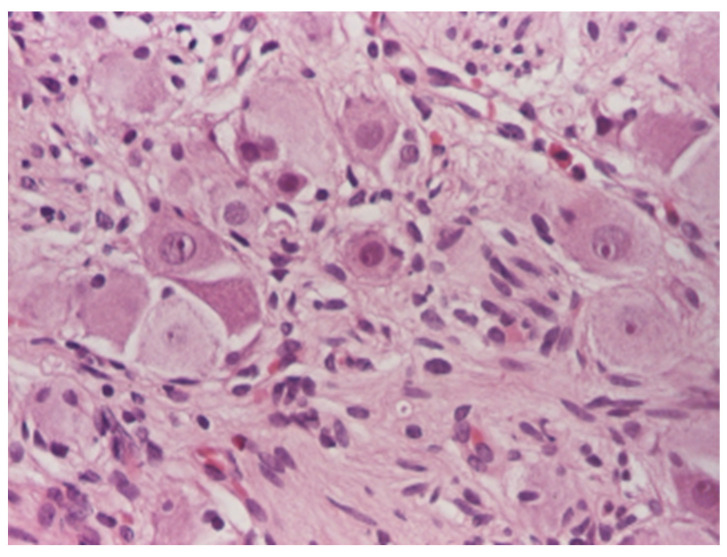
Mature ganglion cells, HE × 200.

Histological assessment: the surgically removed specimens underwent standard processing at the Pathology Laboratory. The samples were fixed in 10% neutral buffered formalin, embedded in paraffin, and sectioned at 3–5 μm. Microscopic examination was performed after tissue staining with Hematoxylin–Eosin (HE) stain and revealed a proliferation of fusiform tumor cells arranged in fascicles with variable orientations associated with mature ganglion cells (Figure 7, Figure 8 and Figure 9). The final diagnosis was bladder ganglioneuroma.

### 2.4. Follow-Up

At the two-year follow-up, the ultrasound and micturition urethrocystography showed no evidence of the progression of the disease, laboratory analyses showed the absence of anemia and urinary tract infection, and the psychological evaluation also highlights a favorable evolution.

## 3. Sources of Information

We used Medical Subject Headings MeSH term “neurofibromatosis type 1” and “bladder ganglioneuroma” and performed a PubMed literature search for systematic reviews, randomized controlled trials, observational studies, and series of cases studies and case reports from the earliest possible date to September 2022. Articles published in English and their reference lists were analyzed for other relevant cases. Searching PubMed for the term “neurofibromatosis type 1”, 13,421 articles were found, and searching for “bladder ganglioneuroma” retrieved 41 articles, but when the terms were combined, only five articles were returned. We reviewed the title and the abstract of the papers and the full text of 51 articles, to extract data on incidence, diagnosis, etiology, associated abnormalities and treatment. Twenty-two of all these articles are found in our reference list.

## 4. Discussion

We presented a case of neurofibromatosis type 1 in an adolescent girl with urinary symptoms associated with a very rare tumor called a bladder ganglioneuroma. The diagnosis of NF1 in the case of our patient was relatively simple, the teenager ticking five of the seven diagnostic criteria. The case was investigated and managed by a multidisciplinary medical team, and the evolution after two years was favorable.

Ganglioneuromas are rare benign tumors which arise from the neural crest and are composed of well-differentiated ganglion cells, nerve fibers, and a large number of Schwann cells. They usually develop in the posterior mediastinum, retroperitoneal space, adrenals and brain, sometimes in the spine, and very rarely in the pelvis. Bladder ganglioneuroma is extremely rare in adults, and only three such cases were reported in children so far [1,5,6]. Ganglioneuromas account for less than 0.5% of all primary bladder tumors in adults and may cause urinary tract obstruction, lower urinary tract symptoms (gross hematuria, urinary tract infection, dysuria), and pelvic pain, as well as hypertension [7]. Sometimes dysfunctional voiding and urinary retention related to mass localization can occur; aggressive surgery and adequate antibiotic therapy is needed [2,8]. Our patient had gross hematuria, complaints of irritative symptoms at the beginning, and urinary tract infections, with good evolution after medical treatment.

In addition to the clinical examination, imaging investigations are to be used to assess the manifestations of neurofibromatosis in the spine, abdominopelvic, and cranial regions [2]. In radiological investigations, bladder involvement is seen as a mass invading the bladder wall or as a better-defined tumor. The CT findings of bladder neurofibroma, ganglioneuromas, or other tumors can be similar and include a lobulated round shape mass with low density and uniform appearance, without increases in density with enhanced CT scan [1,9]. The presented patient showed a large asymmetric and heterogeneous infiltrative tumor in abdominal ultrasound and Uro-CT scans, and even with the help of cystoscopy, the diagnosis of certainty could not be established. Therefore, for establishing the cause of the hematuria in our case, in addition to the imaging investigations, biopsy of the bladder mass was necessary.

Due to the rarity and the histological similarities of each disease, clinical and radiological diagnosis of bladder neurogenic tumors is difficult, especially if needle biopsy is performed [10]. For example, two of three initial cases of ganglioneuroma (needle biopsies) were in fact neurofibroma in a study done by Lee et al. l in 2018 [10]. The rarity of ganglioneuroma tumors and the lack of understanding of their biology may lead to inaccurate diagnosis and treatment [11]. A very precise diagnosis is necessary because the clinical course of each disease is variable, and that is why we performed open biopsies and then partial resection of the tumor with the pathological result being ganglioneuroma; no malignant transformation was detected.

Urogenital involvement (neurofibroma or plexiform neurofibromas) is also very rare in NF1 and only 80 cases have been reported to date in the world literature [2,12]. Our patient had a hypertrophy of the labia majora and a plexiform neurofibroma, which caused her additional distress for cosmetic reasons. Short stature and severe kyphoscoliosis also contributed to low self-esteem, but after psychological counseling she declared an improvement in her quality of life. In the future, inoperable plexiform tumors will be treated with a new drug, Selumetinib, an oral selective mitogen-activated protein kinase (MEK) kinase inhibitor [13,14,15].

Another important problem in patients with NF1 is the risk of developing malignant peripheral nerve sheath tumor later in life of 5 to 10–13%, while it is 0.001% in the general population [16,17]. The molecular characteristics, clinical behavior, and progression of bladder tumors in children are, with the exception of rhabdomyosarcoma, less known than those of adult patients [18], and that is why we chose to present this rare case of an adolescent girl with plexiform neurofibroma and ganglioneuroma. Recurrences of the excised bladder tumors or new tumors may appear later in life, and that is why long-term monitoring is necessary for patients with urologic involvement [19].

Due to the great variability of clinical signs, symptoms, and organ involvement, the patients with NF1 need an individualized treatment and periodic reassessment. We demonstrated in our article this variability of the severity of the disease and the phenotypic aspect, by presenting the case of the sixteen-year-old teenager. Considering the fact that the disease is autosomal dominant and the risk of having offspring with the same condition is 50%, genetic counseling is extremely important in monitoring and preventing the occurrence of serious conditions [20].

Adolescents with unusual diseases may face challenges different from those with frequent medical disorders, especially if they come from disadvantaged social backgrounds or disorganized families [9,21]. The clinical approach of the medical multidisciplinary team involved should help the patient in managing her medical, surgical, and social situations [9,22].

## 5. Conclusions

Due to the variability of clinical manifestations, NF1 represents a therapeutic challenge, most of the times necessitating a multidisciplinary team to develop strategies for diagnosis of multisystem involvement, risk assessment, initiation of targeted therapy, surgery, counseling, and support. Bladder ganglioneuroma is a very rare neurogenic tumor and there are few cases associated with NF1. The majority of cases reported in the literature were treated with local excisions, as in our case.
